# Design Applicable 3D Microfluidic Functional Units Using 2D Topology Optimization with Length Scale Constraints

**DOI:** 10.3390/mi11060613

**Published:** 2020-06-24

**Authors:** Yuchen Guo, Hui Pan, Eddie Wadbro, Zhenyu Liu

**Affiliations:** 1 Changchun Institute of Optics, Fine Mechanics and Physics (CIOMP), Chinese Academy of Science, Changchun 130033, China; guoyuchen15@mails.ucas.edu.cn (Y.G.); panhui13@mails.ucas.ac.cn (H.P.); 2 University of Chinese Academy of Science, Beijing 100049, China; 3 Department of Computing Science, Umeå University, SE-901 87 Umeå, Sweden; eddiew@cs.umu.se

**Keywords:** topology optimization, fluidic flow, length scale control, morphology mimicking filters

## Abstract

Due to the limits of computational time and computer memory, topology optimization problems involving fluidic flow frequently use simplified 2D models. Extruded versions of the 2D optimized results typically comprise the 3D designs to be fabricated. In practice, the depth of the fabricated flow channels is finite; the limited flow depth together with the no-slip condition potentially make the fluidic performance of the 3D model very different from that of the simplified 2D model. This discrepancy significantly limits the usefulness of performing topology optimization involving fluidic flow in 2D—at least if special care is not taken. Inspired by the electric circuit analogy method, we limit the widths of the microchannels in the 2D optimization process. To reduce the difference of fluidic performance between the 2D model and its 3D counterpart, we propose an applicable 2D optimization model, and ensure the manufacturability of the obtained layout, combinations of several morphology-mimicking filters impose maximum or minimum length scales on the solid phase or the fluidic phase. Two typical Lab-on-chip functional units, Tesla valve and fluidic channel splitter, are used to illustrate the validity of the proposed application of length scale control.

## 1. Introduction

With the trend of miniaturization in recent years, Lab-on-a-chip devices have been widely used in the area of the analysis, synthesis, and separations of biofluidics due to the advantages of high efficiency, portability, and low reagent consumption [1]. Many functions of the conventional analytical laboratory can be achieved on a centimeter-level chip, such as injection, mixing, reaction, cleaning, separation, and detection. In recent years, various microfluidic devices have been designed and applied, such as micropumps, microvalves, micromixers, and microchannels [2,3,4,5]. To pursue more functions and higher efficiency, the design and optimization of microfluidic devices is a continuously studied topic.

Borrvall et al. [6] performed topology optimization for fluidic flow governed by the Stokes equation. Their approach was later generalized to the Navier–Stokes equations by Gersborg–Hansen et al. [7] and Olesen et al. [8]. Topology optimization methods have been used to successfully design lab-on-a-chip microfluidic devices, such as microchannel splitters, micropumps, no-moving-part microvalves, and micromixers [9,10,11,12,13,14,15]. The topology optimization model of fluidic flow usually uses a simplified 2D problem. Extruded versions of the 2D optimization results typically comprise the 3D designs to be fabricated. We remark that if the 2D design is stretched so that the 3D flow path has infinite depth, then the full 3D problem describing the fluidic flow reduces to the 2D problem used in the optimization. However, in practice, the depth of the flow channel is not infinite. The limited flow depth and the no-slip condition, which states that the flow speed is zero along the walls, together make the fluidic performance of the 3D model very different from that of the 2D model. This discrepancy significantly limits the application of the obtained 2D results. Some simplification methods have been proposed by build pseudo-3D models in recent years [16,17,18,19], and these research works are mostly focus on heat sinks problem. Zhao et al. [20] impose length scale control on the cooling channel. Based on our previous work [9,14], the length scale control methods are used to design the microfluidic devices with physical constraints (Diodicity for the example of Tesla valve, or the ratio of flowrate at the outlet for the example of splitter) in this paper.

Electric circuit analogy is a method of calculating and designing microchannel networks by analogizing the foundation parameters of the fluid to those of an electrical circuit [21]. When the height of the channel is constant, the hydraulic resistance is linear with the length and non-linear with the width of the channel. Inspired by this, we limit the widths of the microchannels in the optimization process to reduce the difference between the fluidic performance of the 2D and 3D model. Combinations of morphology mimicking filters [22,23,24] impose maximum or minimum length scales on the solid phase or the fluidic phase. The length scale control also ensures the manufacturability of the obtained layout, which avoids unreasonable small-sized islands inside the fluidic channel or thin-sized width of the fluidic channel. This paper is organized as follows—the topology optimization method and electric circuit analogy method are introduced in Section 2; the length scale control method using the morphology-mimicking filters and several numerical results are presented in Section 3; the discussion and conclusion are stated in Section 4.

## 2. Problem Statement

### 2.1. Fluid Flow Model

Based on the continuity assumption, Navier–Stokes equations describe the fluid flow

(1) $$\begin{array}{cc} \rho (u\cdot \nabla )u-\eta \Delta u+\nabla p & =f,\\ \nabla \cdot u & =0, \end{array}$$

where f is the body forces acting on the fluid and u, *p*, ρ, and η are the velocity, pressure, density, and viscosity of the fluid, respectively. To model the fluid–solid interface, we let f be an artificial friction force, similarly as proposed for the Stokes flow by Borrvall et al. [6]. More precisely, the artificial friction force f=−α(γ)u, where α is the impermeability of the artificial porous material and γ is a material indicator function. The function γ varies in the interval [0, 1], where 0 and 1 denote the solid and fluid phase, respectively. The impermeability of the porous material is the interpolation function of the function γ:

(2) $$\alpha \left(\gamma \right)=\alpha_{\max}\frac{q(1-\gamma )}{q+\gamma },$$

where $\alpha_{\max}$ is the impermeability of the solid phase and *q* is a positive value used to adjust the convexity of the interpolation function. To obtain perfect impermeability of the solid no-slip boundary, $\alpha_{\max}$ should be infinite; but a finite number has to be chosen to ensure numerical stability. For the topology optimization of fluid, the aim of the optimization is usually to minimize

(3) $$\Phi =\int_{\Omega }2\eta \epsilon \left(u\right):\epsilon \left(u\right)+\alpha \left(\gamma \right){\left|u\right|}^{2}\;d\Omega ,$$

where $\epsilon \left(u\right)=\left(\nabla u+\nabla u^{T}\right)/2$, |∗| is $L_{2}$ norm. That is, the typical aim is to minimize the viscous dissipation inside the computational domain Ω.

### 2.2. Electric Circuit Analogy Method

**Figure 1:**
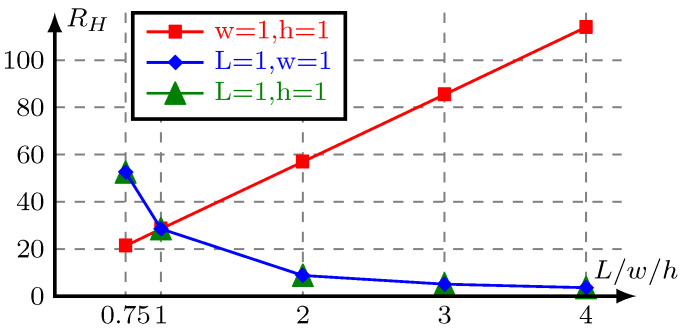
The hydraulic resistance changes with the length (red), height (blue), and width (green) of the channel, η=1.

The electric circuit analogy method provides a fast calculation of laminar flow in microchannels based on the analogous behavior of hydraulic and electric circuits with correlations of pressure to voltage, volumetric flowrate to current, and hydraulic resistance to electric resistance [21]. The hydraulic resistance is an important parameter in the design of microfluidic networks by electric circuit analogy. The hydraulic resistance $R_{H}$ of a rectangular microchannel section can be calculated as [25]

(4) $$R_{H}=\frac{12\eta L}{wh^{3}\left(1-\frac{h}{w}\left(\frac{192}{\pi^{5}}\sum_{n=1,3,5}^{\infty }\frac{1}{n^{5}}\tanh\left(\frac{n\pi w}{2h}\right)\right)\right)},$$

where η is the viscosity of the fluid and *w*, *h*, and *L* are the width, height, and length of the microchannel section, respectively. Figure 1 illustrates the hydraulic resistance changes with the length (red), height (blue), and width (green) of the channel, where the viscosity of the fluid η is normalized as 1. When the height of the channel is constant, the hydraulic resistance changes linearly with the length and nonlinearly with the width of the channel. By limiting the width of the flow channel, the hydraulic resistance changes linearly with the length. The hydraulic resistance ratio between different parts of the fluid network can remain unchanged in different stretching heights, so the difference of fluidic performance between the 2D model and its 3D counterpart is small. In order to preserve the flexibility of topology optimization, the changes of the channel width is constrained within a certain range.

## 3. Numerical Experiments

To demonstrate the validity of the proposed method, which can reduce the difference of fluidic performance between the 2D model and its 3D counterpart by using the size control of the widths of the fluidic channels, two examples are used in this paper. The viscosity and density of the fluid are $10^{-3}$ Pa·s and 998 kg·$m^{-3}$, respectively. The inlet velocity is chosen to have a parabolic profile. The intermediate fluid velocity in the height direction at the entrance of the 3D model is selected to be consistent with the 2D model’s inlet velocity. For all numerical experiments, Svanberg’s method of moving asymptotes [26] solves the resulting optimization problem.

We discretize our computational domain Ω by *N* quadrilateral elements. Comsol multiphysics solves the governing Navier–Stokes equation using bi-linear tensor product elements together with GLS (Galerkin least squares) streamline diffusion with the crosswind diffusion parameter set to 0.1.

**Figure 2:**
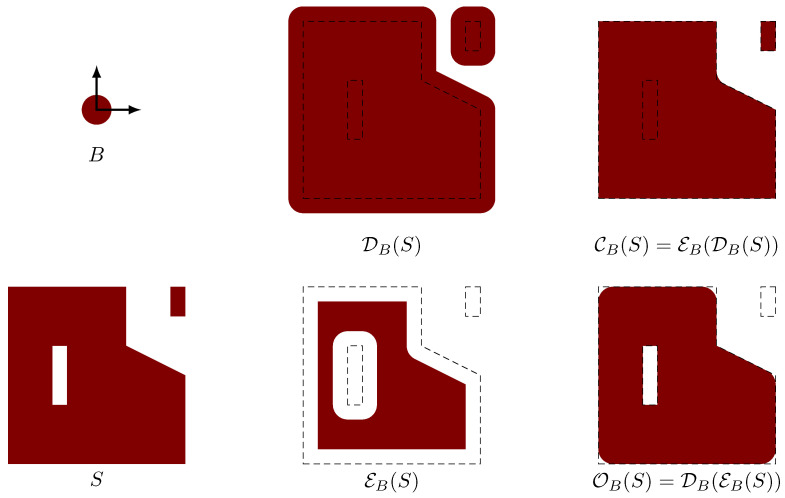
The four basic morphological operators defined by the ball *B* acting on the set *S*.

We let *M* denote the number of nodal points in the discretization of the design domain $\Omega_{D}$. We define our material indicator function γ to be an nodal-wise bi-linear function whose restriction to $\Omega \backslash \Omega_{D}$ satisfies $\gamma {|}_{\Omega \backslash \Omega_{D}}\equiv 1$. The *M* nodal values of the material indicator function in $\Omega_{D}$ are obtained by using a harmonic mean based open–close fW-mean filter. For a comprehensive review of mathematical morphology, we refer the reader to the Heijmans article [27]. Figure 2 illustrates the four-basic morphological operators: dilation, erosion, opening, and closing. In colloquial terms, given a brush with shape *B*, then the opening of *S* by *B* holds all points the can be filled by using this brush without touching any point outside *S*; and given a fully filled space and an eraser with shape *B*, the closing of *S* is the region that remains after having erased as much as possible without removing any point in *S*. Or, in other words, applying the opening operator to a set *S* removes all small parts from *S*; similarly, applying the closing operator to a set *S* removes all small internals holes. The region obtained by the open operation can be written as a union of translates of *B*, and the complement of the region obtained by the close operation can be written as a union of translates of *B*. It can be shown that the minimum size of $O_{B}\left(S\right)$ as well as $R^{d}\backslash C_{B}\left(S\right)$ is at least the size of *B*.

The use of a cascade of filters to approximate the open–close operator was suggested by Sigmund [28], and harmonic mean based filters were introduced by Svanberg et al. [29]. Using the notation for fW-filters [30], we define the equally weighted discrete harmonic erode and dilate operators with radius r>0 and parameter β>0 by

(5) $$\begin{array}{cc} E_{r,\beta }^{H}\left(x\right) & =f_{E_{\beta }^{H}}^{-1}\left(D_{r}^{-1}G_{r}f_{E_{\beta }^{H}}\left(x\right)\right), \end{array}$$

(6) $$\begin{array}{cc} D_{r,\beta }^{H}\left(x\right) & =f_{D_{\beta }^{H}}^{-1}\left(D_{r}^{-1}G_{r}f_{D_{\beta }^{H}}\left(x\right)\right), \end{array}$$

where x is an M×1 vector; $f_{E_{\beta }^{H}}\left(x\right)$ and $f_{D_{\beta }^{H}}\left(x\right)$ represent entry-wise application of the functions $f_{E_{\beta }^{H}}$ and $f_{D_{\beta }^{H}}$, defined as $f_{E_{\beta }^{H}}\left(x\right)={\left(x+\beta \right)}^{-1}$ and $f_{D_{\beta }^{H}}\left(x\right)=f_{E_{\beta }^{H}}\left(1-x\right)$, respectively; the neighborhood matrix $G_{r}$ has entries $g_{ij}=1$ if the distance between the centroids of elements *i* and *j* is smaller than *r*, else $g_{ij}=0$; and $D_{r}=\mathrm{diag}\left\{G_{r}1\right\}$, where 1 is the M×1 vector with all entries set to one. The vector γ that holds the nodal values of the material indicator function γ in $\Omega_{D}$ is

(7) $$\gamma =E_{r_{c},\beta }^{H}\left(D_{r_{c},\beta }^{H}\left(D_{r_{o},\beta }^{H}\left(E_{r_{o},\beta }^{H}\left(\xi \right)\right)\right)\right),$$

where ξ is our design vector. In the numerical experiments, we use parameter $\beta =10^{-4}$. The values for $r_{o}$ and $r_{c}$ dictate the desired minimum length scale for the fluidic and solid phase, respectively. These values are selected separately for each experiment. We remark that the existence of a minimum size scale for both phases cannot be guaranteed when using open–close filters [31]. However, in many cases the optimized designs, obtained by using such filters, possess a minimum size scale on both phases [24,28].

To impose a constraint on the total volume of the fluidic phase as well as to constrain the maximum length scale of the fluidic phase, we add additional two constraints [22,23]

(8) $$\begin{array}{cc} v^{T}\gamma & \leq V^{*}, \end{array}$$

(9) $$\begin{array}{cc} v^{T}E_{r_{m},\beta }^{H}\left(\gamma \right) & \leq \varepsilon , \end{array}$$

where $V^{*}$ is the maximum fraction of Ω to be filled with the fluid, $\varepsilon =10^{-4}$, parameter $r_{m}$ specifies the desired maximum length scale of the fluidic channels, and v is a vector with entries

(10) $$v_{i}=\frac{1}{|\Omega_{D}|}\int_{\Omega_{D}}\varphi_{i}\;d\Omega ,$$

where $\varphi_{i}$ is basis function corresponding to the *i*th node in $\Omega_{D}$ and $|\Omega_{D}|$ is the area of $\Omega_{D}$.

### 3.1. Tesla Valve

**Figure 3:**
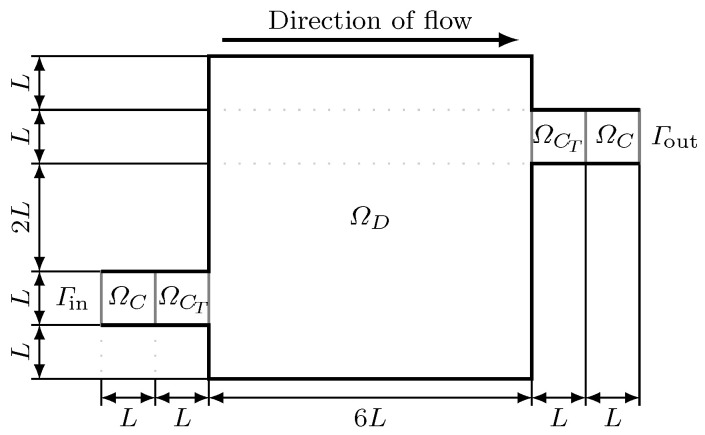
The computational domain Ω for the Tesla valve.

The Tesla valve is a basic device for controlling fluid motion [14,32]. Figure 3 shows the computational domain Ω for the Tesla valve. We denote the channels connected to the inlet and outlet by $\Omega_{C}$, and the Tesla valve occupies the domain $\Omega_{T}=\Omega_{C_{T}}\cup \Omega_{D}$, where $\Omega_{D}$ is the design domain. The width of inlet *L* is 200 μm.

The domain for the Tesla valve is a two-port network, where boundaries $\Gamma_{\mathrm{in}}$ and $\Gamma_{\mathrm{out}}$ represent the two ports. We consider the flow in the two directions from $\Gamma_{\mathrm{in}}$ to $\Gamma_{\mathrm{out}}$ and vice-versa. Henceforth, we denote the two flow directions by the forward and backward direction, respectively. In both the forward and backward direction, the flow profile is specified at the port from which the fluid flows. We let $p_{f}$ and $u_{f}$ denote the pressure and velocity, respectively, for the forward-directed fluid field. Similarly, $p_{r}$ and $u_{r}$ denote the pressure and velocity, respectively, for the backward-directed fluid field. The forward flow can be modeled by Navier–Stokes Equation (1) in Ω together with the boundary condition

(11) $$\begin{array}{cccc} u_{f} & =u_{0} & \; & \mathrm{on}\;\Gamma_{\mathrm{in}},\\ \left[-p_{f}I+2\eta \epsilon \left(u_{f}\right)\right]\cdot n & =0 & \; & \mathrm{on}\;\Gamma_{\mathrm{out}}, \end{array}$$

where $u_{0}$ is the given flow velocity profile at $\Gamma_{\mathrm{in}}$. The backward flow can be modeled by Navier–Stokes Equation (1) in Ω together with the boundary condition

(12) $$\begin{array}{cccc} u_{r} & =u_{0} & \; & \mathrm{on}\;\Gamma_{\mathrm{out}},\\ \left[-p_{r}I+2\eta \epsilon \left(u_{r}\right)\right]\cdot n & =0 & \; & \mathrm{on}\;\Gamma_{\mathrm{in}}, \end{array}$$

where $u_{0}$ is the given flow velocity profile at $\Gamma_{\mathrm{out}}$. The performance of Tesla valve is measured by diodicity, which is defined as the ratio of pressure drop of the forward flow to that of the reverse flow. From the kinetic energy viewpoint, the diodicity is also expressed as the ratio of the consumption of the forward pressure work to the reverse pressure work, under the assumption that the flux in the two opposite directions is equal. The diodicity of the Tesla valve can be alternatively calculated as [13,14,32,33]

(13) $$\mathrm{Di}=\frac{-\int_{\Gamma_{\mathrm{in}}}p_{f}\left(u_{f}\cdot n\right)\;d\Gamma -\int_{\Gamma_{\mathrm{out}}}p_{f}\left(u_{f}\cdot n\right)\;d\Gamma }{-\int_{\Gamma_{\mathrm{in}}}p_{r}\left(u_{r}\cdot n\right)\;d\Gamma -\int_{\Gamma_{\mathrm{out}}}p_{r}\left(u_{r}\cdot n\right)\;d\Gamma }=\frac{\int_{\Omega_{T}}\nabla \cdot \left(p_{f}u_{f}\right)\;d\Omega }{\int_{\Omega_{T}}\nabla \cdot \left(p_{r}u_{r}\right)\;d\Omega }.$$

The topology optimization problem is

(14) $$\begin{array}{cccc} \min_{\xi \in R^{M}} & \; & \Phi ,\\ \mathrm{subject}\;\mathrm{to} & \; & \mathrm{Di} & \leq C_{\max},\\ \; & \; & v^{T}\gamma & \leq V^{*},\\ \; & \; & 0\leq \xi & \leq 1. \end{array}$$

**Figure 4:**
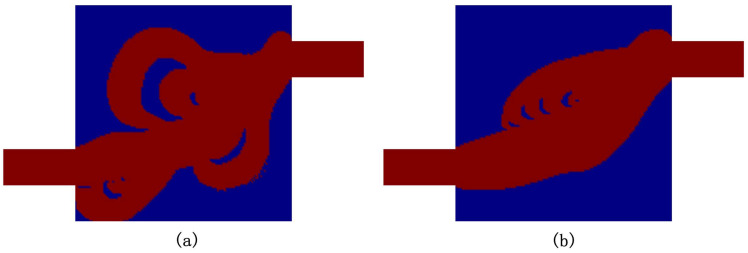
Two optimized Tesla valves with the value of $C_{\max}$ is 0.351 (**a**) and 0.551 (**b**), respectively.

For the numerical simulations, each square space with an edge size equal to *L* is discretized by 20×20 quadrilateral elements. Hence 120×120 elements discretize the design domain $\Omega_{D}$. The values of $\alpha_{\max}$ and *q* in the topology method are chosen to be $5\times 10^{8}$ and 1, respectively. Here, we set length scale parameters $r_{c}$ and $r_{o}$ small enough to ensure that the neighborhood of each node in $\Omega_{D}$ only consists of the node itself. This yields that γ=ξ. The initial value of the design variable ξ is 1, the Reynolds number is 100, and $V^{*}$ is 0.55. Figure 4 shows two optimized Tesla valves. Due to physical problems and geometric constraints, there is a lower limit for the diodicity of the Tesla valve in a particular design domain. And the optimization results depend on the optimization process. Initially, we set a low $C_{\max}$ and then increased during the optimization. The left and right valves are obtained for the value of $C_{\max}$ is 0.351 and 0.551, respectively. We create body-fitted 2D models that only consider the fluidic phase. The diodicities computed by using these body-fitted 2D models are 0.408 and 0.600 for the left and right design in Figure 4, respectively.

Next, we add a constraint on the maximum length scale in the optimization model and consider the problem

(15) $$\begin{array}{cccc} \min_{\xi \in R^{M}} & \; & \Phi ,\\ \mathrm{subject}\;\mathrm{to} & \; & \mathrm{Di} & \leq C_{\max},\\ \; & \; & v^{T}\gamma & \leq V^{*},\\ \; & \; & v^{T}E_{r_{m},\beta }^{H}\left(\gamma \right) & \leq \varepsilon ,\\ \; & \; & 0\leq \xi & \leq 1. \end{array}$$

**Figure 5:**
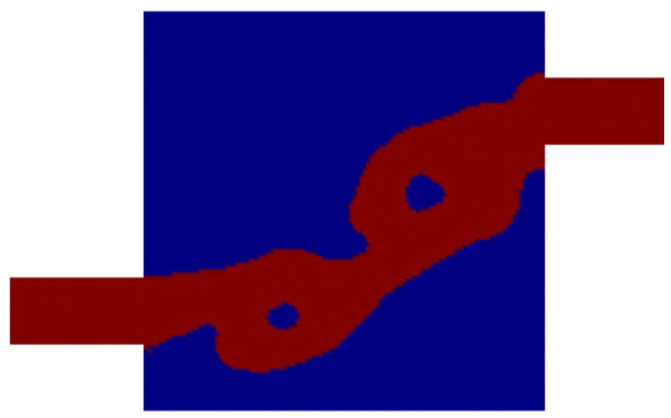
Tesla valve optimized with the full length scale control optimization Formulation (15).

The maximum and minimum length of the fluidic phase are $r_{m}$ = 200 μm and $r_{o}$ = 100 μm, respectively, and the minimum length of the solid phase is $r_{c}$ = 60 μm. For this test, we set $V^{*}=0.25$ and $C_{\max}=0.5$. Figure 5 shows the optimized Tesla valve with the value of $C_{\max}=0.829$. We can see that the optimized design is very similar to the two commonly used Tesla valves in series. We create a body-fitted 2D model that only considers the fluidic phase. The diodicity computed by using the body fitted 2D model is 0.882. It is clear that the design constraint of diodicity is violated because of using size control during the optimization procedure.

**Figure 6:**
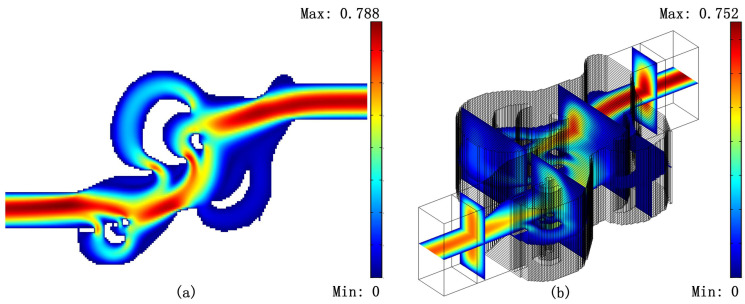
Reverse flow velocity field of the 2D model (**a**) and its 3D counterpart (**b**) for the optimized Tesla valve with the value of $C_{\max}=0.408$ in Figure 4.

**Figure 7:**
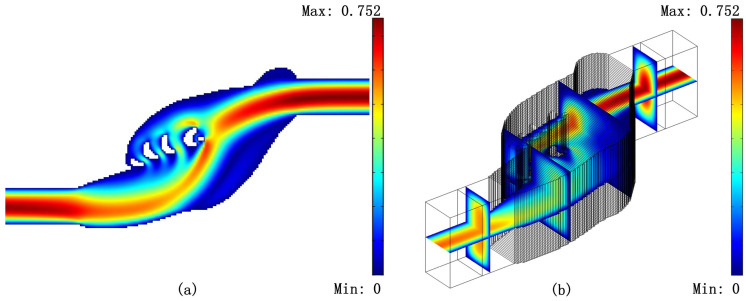
Reverse flow velocity field of the 2D model (**a**) and its 3D counterpart (**b**) for the optimized Tesla valve with the value of $C_{\max}=0.600$ in Figure 4.

**Figure 8:**
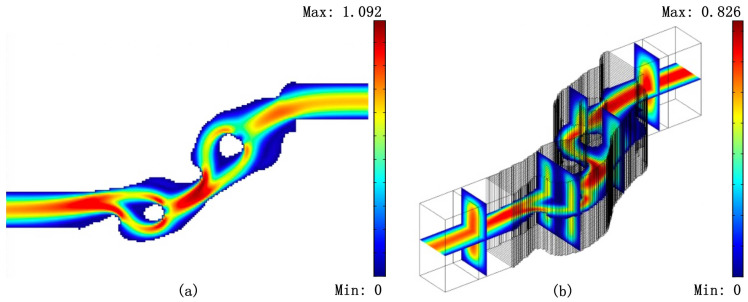
Reverse flow velocity field of the 2D model (**a**) and its 3D counterpart (**b**) for the optimized Tesla valve in Figure 5.

**Figure 9:**
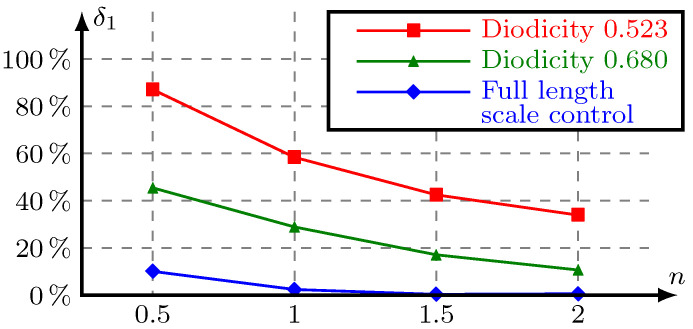
The difference $\delta_{1}$ of three optimization results between 2D and 3D models with the ratio of depth to width of the inlet *n*.

Finally, we create 3D models for the results in Figure 4 and Figure 5 by stretching the body-fitted 2D models. Figure 6, Figure 7 and Figure 8 shows the velocity fields of the 2D model (left image) as well as the 3D model (right image) for the optimized Tesla valve in Figure 4 and Figure 5. Having access to the 3D models, we compute the difference

(16) $$\delta_{1}=\left|1-\frac{{\mathrm{Di}}_{3D}}{{\mathrm{Di}}_{2D}}\right|\times 100\;\%,$$

where the subscripts 2D and 3D represent the 2D model and its 3D counterpart, respectively. In the ideal case, $\delta_{1}$ is equal to 0. Figure 9 shows the difference of three optimization results between 2D and 3D models with the ratio of depth to width of the inlet n. The difference in fluidic performance between the optimized 2D model and its 3D counterpart gradually decreases as the height of the 3D model increases. This confirms that the 2D model assumption that the height is infinite, the higher the stretching height of the 3D model, the smaller the difference between the 2D model and the 3D model. The difference of the optimization results using dimension control is significantly smaller than that of unused optimization results, and it is acceptable when *n* is greater than 1.

### 3.2. Microfluidic Splitters with Equivalent Outlet Flowrate

**Figure 10:**
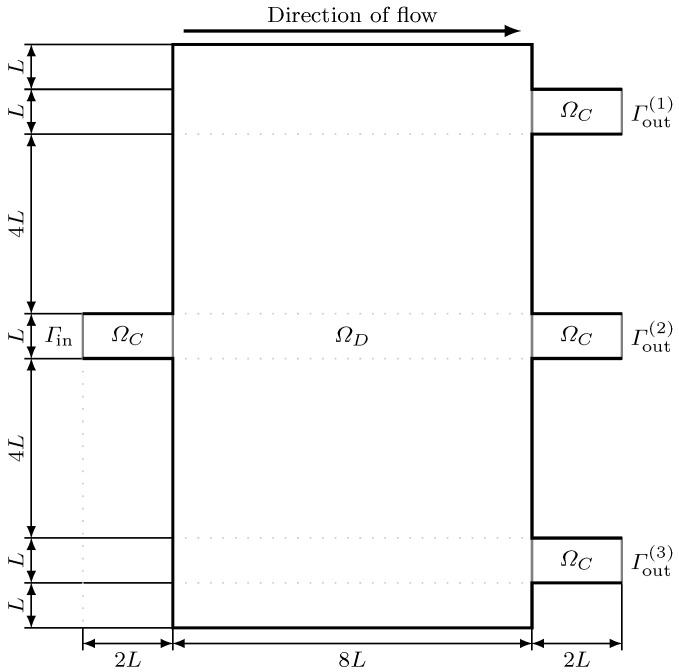
Computational domain for the second test problem. Here, the computational domain Ω comprises the design domain $\Omega_{D}$ as well as four channels $\Omega_{C}$ that are connected to the inlet and outlets of the domain.

As our second test problem, we study the problem of designing microfluidic splitters originally proposed by Zhou et al. [9]. Figure 10 shows the computational domain $\Omega =\Omega_{C}\cup \Omega_{D}$ for this test case. The width of inlet *L* is 100 μm. Here, we have one inlet $\Gamma_{\mathrm{in}}$ and three outlets $\Gamma_{\mathrm{out}}^{\left(1\right)}$, $\Gamma_{\mathrm{out}}^{\left(2\right)}$, and $\Gamma_{\mathrm{out}}^{\left(3\right)}$; we denote the union of the outlets by $\Gamma_{\mathrm{out}}$. Given a specified velocity profile, given by $u_{0}$ on the inlet, the flow is modeled by Navier–Stokes Equation (1) in Ω together with boundary condition (11).

In this example, we consider two fluidic cases in a four-port splitter. The flow direction is from $\Gamma_{\mathrm{in}}$ to $\Gamma_{\mathrm{out}}$ in both cases. In contrast to the previous example, we will require a specified flowrate,

(17) $$Q=\int_{\Gamma }u\cdot n\;d\Gamma ,$$

at selected ports of the domain. We let $p_{\mathrm{real}}$ and $u_{\mathrm{real}}$ denote the pressure and velocity, respectively, for the so-called (in this work) real case. The real case is modeled by Navier–Stokes Equation (1) in Ω together with the boundary condition

(18) $$\begin{array}{cccc} \int_{\Gamma_{\mathrm{in}}}u_{\mathrm{real}}\cdot n\;d\Gamma & =Q_{\mathrm{in}} & \; & \mathrm{on}\;\Gamma_{\mathrm{in}},\\ \left[-p_{\mathrm{real}}I+2\eta \epsilon \left(u_{\mathrm{real}}\right)\right]\cdot n & =0 & \; & \mathrm{on}\;\Gamma_{\mathrm{out}}. \end{array}$$

Here $Q_{\mathrm{in}}$ is the given flowrate at $\Gamma_{\mathrm{in}}$. Similarly, we let $p_{\mathrm{ref}}$ and $u_{\mathrm{ref}}$ denote the pressure and velocity, respectively, for the so-called reference case. The reference case is modeled by Navier–Stokes Equation (1) in Ω together with the boundary condition

(19) $$\begin{array}{cccc} \left[-p_{\mathrm{ref}}I+2\eta \epsilon \left(u_{\mathrm{ref}}\right)\right]\cdot n & =0 & \; & \mathrm{on}\;\Gamma_{\mathrm{in}},\\ \int_{\Gamma_{\mathrm{out}}^{\left(i\right)}}u_{\mathrm{ref}}\cdot n\;d\Gamma & =-Q_{\mathrm{in}}/3 & \; & \begin{array}{c} \mathrm{on}\;\Gamma_{\mathrm{out}}^{\left(i\right)}\\ \mathrm{for}\;i=1,2,3. \end{array} \end{array}$$

The topology optimization problem is

(20) $$\begin{array}{cc} \min_{\xi \in R^{M}}\; & \frac{\Phi_{\mathrm{real}}+\Phi_{\mathrm{ref}}}{b+1-v^{T}\gamma }+\theta \left(\Phi_{\mathrm{real}}-\Phi_{\mathrm{ref}}\right)\\ \mathrm{subject}\;\mathrm{to}\; & 0\leq \xi \leq 1. \end{array}$$

**Figure 11:**
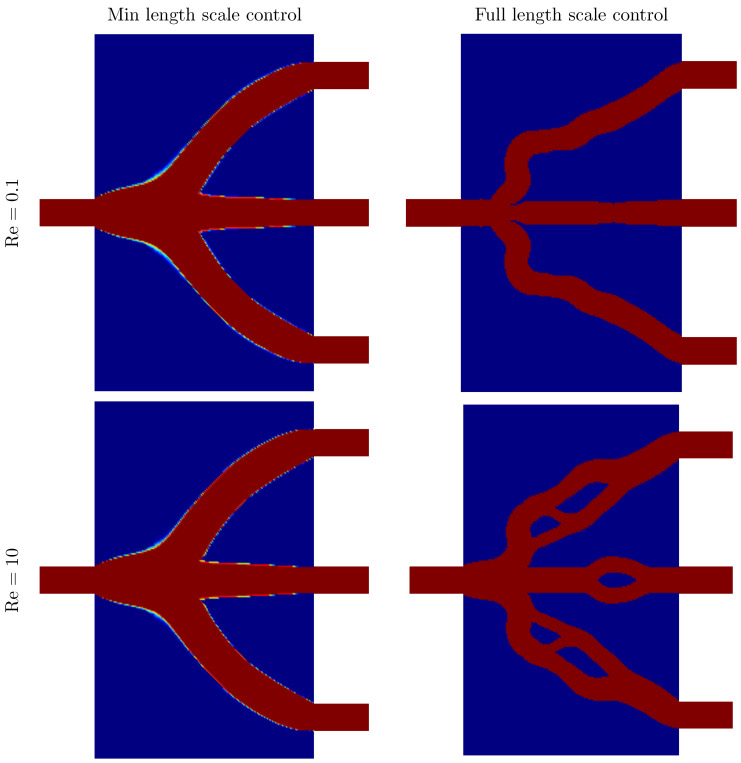
Optimized designs with aim to obtain equivalent outlet flowrate.

The first term of the objective function is a fraction, in which the numerator is the sum of the viscous dissipations of the two cases and the denominator is the volume of the solid phase, to obtain an appropriate volume automatically. The second term of the objective function is the difference between the viscous dissipations of the two cases, and θ is the scalar number used to penalize this difference. By this method, the flowrate constraint of outlet in the real model is realized. The values of $\alpha_{\max}$ and *q* in the topology method are $5\times 10^{9}$ and 1, respectively. The initial value of the design variable ξ is 0.5. During the optimization, θ increases from 0 to 100. The left column of Figure 11 shows designs optimized without length scale control. That is, just as for the results in Figure 4, $r_{c}$ and $r_{o}$ are small enough to ensure γ=ξ. The Reynolds numbers for the simulations were 0.1 (top row), and 10 (bottom row), and the value of *b* in the objective function was 0.1.

The right column of Figure 11 shows designs optimized under a maximum length scale constraint for the fluid domain. That is, we solve optimization problem (20) appended with constraint (9).

The maximum length of the fluidic phase is 100 μm, and the value of *b* used in the objective function is 0.1 and 0.2 for Reynolds numbers 0.1 and 10, respectively.

**Figure 12:**
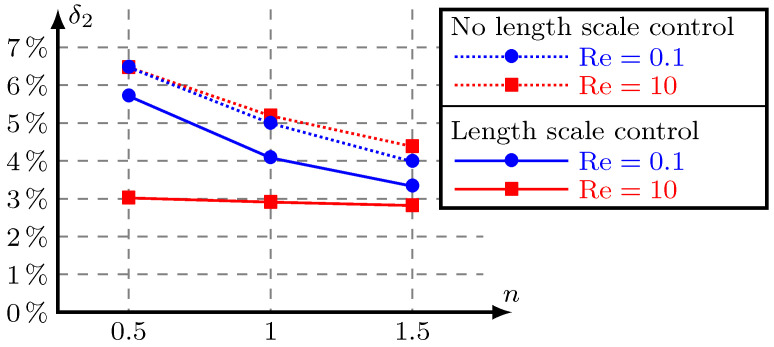
The difference $\delta_{2}$ of four optimization results between 2D and 3D models with the ratio of depth to width of the inlet *n*.

Having access to the 3D models, we compute the difference

(21) $$\delta_{2}=\frac{1}{3}\sum_{i=1}^{3}\left|1-\frac{3Q^{\left(i\right)}}{Q^{\left(1\right)}+Q^{\left(2\right)}+Q^{\left(3\right)}}\right|\times 100\;\%,$$

where $Q^{\left(i\right)}$ is the flowrate at port $\Gamma_{\mathrm{out}}^{\left(i\right)}$. In the ideal case, $\delta_{2}$ is equal to 0. Figure 12 shows the difference for 3D models corresponding to optimized designs in Figure 11 as functions of the ratio of depth to width of the inlet n. The difference in fluidic performance between the optimized 2D model and its 3D counterpart gradually decreases as the height of the 3D model increases. The difference of the optimization results using dimension control is significantly smaller than that of unused optimization results when Re = 10. and it is not significantly smaller than that of unused optimization results when Re = 0.1. The value of Reynolds numbers maybe influences the effect of the proposed method.

## 4. Discussion and Conclusions

We can see that the difference in fluidic performance between the optimized 2D model and its 3D counterpart gradually decreases as the height of 3D model increases in Figure 9 and Figure 12. This conforms to the 2D model assumption is that the height is infinite.

There is a significant difference in fluidic performance in the case where no length scale control is applied. The difference in the optimization result of length scale control applied is much smaller. Imposing size control of the widths of the fluidic channels can effectively reduce the difference of fluidic performance between the 2D model and the 3D model obtained by stretching the 2D model, so that the 2D optimization results of flow have more practical significance. Moreover, the length scale control also ensures the manufacturability of the obtained. Numerical results demonstrate the validity of the proposed method. We hope that this paper will spur further interest and development on this topic.
